# SARS-CoV-2 Seroprevalence in Delhi, India, During September-October 2021: A Population-Based Seroepidemiological Study

**DOI:** 10.7759/cureus.27428

**Published:** 2022-07-28

**Authors:** Pragya Sharma, Saurav Basu, Suruchi Mishra, Ekta Gupta, Reshu Agarwal, Pratibha Kale, Nutan Mundeja, BS Charan, Gautam Singh, Mongjam Singh

**Affiliations:** 1 Community Medicine, Maulana Azad Medical College, New Delhi, IND; 2 Epidemiology and Public Health, Indian Institute of Public Health, Public Health Foundation of India, New Delhi, IND; 3 Community Medicine, Vardhaman, New Delhi, IND; 4 Virology, Institute of Liver and Biliary Sciences, New Delhi, IND; 5 Microbiology, Institute of Liver and Biliary Sciences, New Delhi, IND; 6 Health Services, Government of National Capital Territory, New Delhi, IND; 7 Preventive Medicine, Directorate General of Health Services, Government of National Capital Territory, Delhi, IND; 8 State Surveillance Unit, Directorate General of Health Services, Government of National Capital Territory, Delhi, New Delhi, IND

**Keywords:** india, vaccination, covid-19, seroprevalence, serosurvey

## Abstract

Background

A previous community-based severe acute respiratory syndrome coronavirus 2 (SARS-CoV-2) serosurvey in Delhi in January 2021 reported a seroprevalence of 50.52%. We conducted a repeat serosurvey to obtain a recent estimate of the seroprevalence of IgG SARS-CoV-2 in the general population of Delhi, India.

Methods

This cross-sectional study was conducted from September 24 to October 14, 2021, in 274 wards of Delhi among 27,811 participants through a multistage sampling technique.

Results

The crude seroprevalence was 89.5% (95% CI 89.1, 89.8), weight for age and sex was 88% (95% CI 87.6, 88.4), and after adjustment for assay performance was estimated as 97.5% (95% CI 97.0, 98.0). On adjusted analysis, the odds of seroconversion in the participants vaccinated with at least one dose of either COVID-19 vaccine (Covishield/Covaxin) was more than four times compared to the unvaccinated ones (aOR 4.2 (3.8, 4.6)). 86.8% of the seropositive individuals had a SARS-CoV-2 signal/cut-off ≥4.0 although it was significantly lower in the pediatric age group. Post-second wave (August to October 2021), on average there were daily 39 new COVID-19 cases and 0.44 deaths which during Omicron driven the third wave in January to March 2022 increased to daily 4,267 cases and 11.6 deaths.

Conclusion

A high prevalence of IgG antibodies against SARS-CoV-2 with likely higher antibody titres in the vaccinated compared to the unvaccinated groups with evidence of hybrid immunity in a majority of the population was protective against severe disease during transmission of subsequent omicron variants.

## Introduction

The National Capital Territory of Delhi experienced a severe second wave of COVID-19, predominantly driven by the Delta variant during April-June 2021 [1,2]. The city, having a population of ~19 million, had recorded ~1.83 million cases and 26,158 deaths as of April 14, 2022 [3].

COVID-19 seroprevalence studies estimate the population level humoral immunity profile by direct measurement of SARS-CoV-2 antibodies induced through either natural infection or vaccination [4]. Repeated cross-sectional seroepidemiological studies when regionally localized enable seroprevalence monitoring for guiding infection prevention and control strategies by mounting effective public health interventions [5].

Serial serosurveys have previously indicated that the age and sex weighted seroprevalence in Delhi in the general population increased from 24.71% (95% CI 24.01 to 25.42) in August-October 2020 to 50.52% (95% CI 49.94 to 51.10 in January 2021) [6,7].

India’s COVID-19 vaccination campaign was formally initiated on January 16 2021 and was predominantly driven by ChAdOx1 nCoV-19 (Covishield, Serum Institute of India, Pune) and BBV152 (Covaxin; Bharat Biotech International, Hyderabad). The vaccination strategy was initially confined to healthcare and frontline workers (January 16, 2021 onwards), was subsequently expanded to the elderly (>60 years old) and select comorbid (>45 years old) individuals (March 1, 2021 onwards), followed by all >45 years old (April 1, 2021 onwards), and lastly all individuals >18 years old (May 1 2021 onwards). Vaccination in the 15-18 age group was initiated in January 2022. As of October 21, 2021, 19.8 million cumulative vaccine doses were administered to the eligible beneficiaries [8].

We conducted this sixth round of Delhi state serosurvey to estimate the seroprevalence of SARS-CoV-2 in the general population and compare the antibody prevalence in the vaccinated and non-vaccinated groups.

This article was previously posted to the medRxiv preprint server on December 29, 2021.

## Materials and methods

Study design, participants and settings

This was a cross-sectional, seroepidemiological study among individuals aged five and above who were selected from 274 wards in the state of Delhi from September 24, 2021 to October 14, 2021. Children below five years were excluded from the study due to the inherent phlebotomy challenges in the under-5 group, the requirement for diversion of highly trained personnel from routine medical duties, and the parental hesitancy in subjecting their children to phlebotomy that was not necessary. 

A total of 100 participants each were enrolled from 274 wards except the Delhi Cantonment and the New Delhi wards which had larger populations. It was estimated that the sample size of approximately 28,000 was adequate at 99% confidence levels, 1% absolute precision, 56% expected prevalence from the previous serosurvey [7], design effect of 1.5, and considering a non-response rate of 10%.

The sampling was conducted within each ward based on the residential settlement types which included planned colonies, urban slums, resettlement colonies, unauthorized colonies, and rural areas [9]. Within each ward, the proportion of participants selected from each settlement type was stratified according to their tentatively estimated population size. The participants were selected for this household serosurvey through a multistage sampling technique having the following steps: (i) Selection of the sampling areas within each settlement type by simple random sampling, (ii) household selection by systematic random sampling, and (iii) age-order procedure for selection of an individual within each selected household.

Laboratory procedure

A trained phlebotomist cum laboratory technician collected 3-4 mL of a venous blood sample under aseptic precautions which were then transported and processed at the designated laboratory at the Department of Virology, Institute of Liver and Biliary Sciences, New Delhi. Anti-SARS-CoV-2 IgG antibodies were detected using chemiluminescent technology-based VITROS® assay on a single integrated platform of VITROS® 3600 (Ortho Clinical Diagnostics, Raritan, NJ, USA) immunodiagnostic system [10]. The assay has a documented sensitivity of 90% and specificity of 100% which is deemed acceptable for SARS-CoV-2 seroprevalence surveys [11]. Furthermore, 10% of the samples were randomly assessed for the presence of SARS-CoV-2 neutralizing antibodies that indicated a strong correlation with an IgG signal/cut-off ratio ≥4 [12].

Statistical analysis

A customized android tablet application was used for electronic data collection by field volunteers recruited for this survey. The variables included sociodemographic data, past history of COVID-19 disease, and COVID-19 vaccination status. This questionnaire data were subsequently merged with the laboratory antibody test result data using Microsoft Excel 2013 software with the VLOOKUP command. The data were analysed using SPSS Version 25 (IBM Corp., Armonk, NY, USA). The seroprevalence estimates were weighted to match the territorial demographics by age and sex and reported as proportions with 95% confidence intervals (CIs). The adjusted seroprevalence was estimated through statistical correction of the weighted seroprevalence by incorporating the assay characteristics in the Rogan-Gladen estimator, where true (adjusted) prevalence = weighted prevalence + (specificity - 1)/(specificity + sensitivity - 1) [13]. Results were expressed as frequency and proportions for categorical variables and mean and standard deviation for continuous variables. The chi-square test was used to assess the association among categorical variables. A binary logistic regression analysis was conducted by considering IgG antibody as the outcome and the following independent variables; sex: male/female, age: <18/≥18, settlement: planned/other, past-history of COVID-19: present/absent, and vaccination status: no dose/at-least one dose. A p-value of <0.05 was considered statistically significant.

## Results

A total of 28,491 laboratory samples were collected, out of which 27,811 were successfully processed in the laboratories. There were 24,895 (89.5%) samples detected having IgG SARS-CoV-2 antibodies. The crude seroprevalence was 89.5% (95% CI 89.1, 89.8). The seroprevalence weighted for age and sex was 88% (95% CI 87.6, 88.4). The weighted seroprevalence in the districts ascended from 84.9% (South-West district), 85.8% (Shahdara), 86.5% (North), 87.0% (New Delhi), 87.2 (Southeast), 87.5% (West), 89.6% (Northwest), 90.0% (South), 90.5% (Central), 90.7% (North East) to 90.8% (East) district. The adjusted seroprevalence estimated after statistical correction of the weighted seroprevalence for assay characteristics was 97.5% (95% CI 97.0, 98.0). On bivariate analysis, females in all the age-groups (<18, 18-49 and ≥50) had significantly higher odds of seropositivity than males (p<0.001) (Table 1).

**Table 1:** Age and sex-stratified seroprevalence of antibodies to SARS-CoV-2, Delhi, September-October 2021

Sex	Total (n=27811)	IgG Seropositive	IgG Seronegative	Adjusted odds (95% CI)
Male				
<18	2,165 (18.4)	1,765 (81.5)	400 (18.5)	1 (p<0.001)
18-49	6,838 (58.0)	6,147 (89.9)	691 (10.1)	2.0 (1.76, 2.3)
≥50	2,788 (23.6)	2,489 (89.3)	299 (10.7)	1.88 (1.6, 2.21)
Female				
<18	2,046 (12.8)	1,680 (82.1)	366 (17.9)	1 (p<0.001)
18-49	10,357 (64.7)	9,388 (90.6)	969 (9.4)	2.11 (1.85, 2.4)
≥50	3,617 (22.5)	3,426 (94.7)	191 (5.3)	3.9 (3.25, 4.7)

Our logistic regression model was statistically significant with Chi^2^(5) = 1,105.5 (p<0.001) and correctly classified 89.3% of the cases. The Hosmer Lemeshow goodness of fit test statistic had a p-value of 0.124 from which it was concluded that the model estimates the data acceptably. Based on this adjusted analysis, we found the participants of the female gender (aOR 1.3 [1.2, 1.4]), and those having received at least one dose of either of the two COVID-19 vaccines (aOR 4.2 [3.8, 4.6]) as statistically significant predictors of SARS-CoV-2 seropositivity (p<0.001) (Table 2).

**Table 2:** Seroprevalence of antibodies to SARS-CoV-2, Delhi, September-October 2021 +Missing sociodemographic data for 3,631 participants

Characteristic	Total (n=24180)^+^	IgG Seropositive	IgG Seronegative	Adjusted odds (95% CI), p
Sex				
Male	10,231 (42.3)	9,006 (88)	1,225 (12)	1
Female	13,949 (57.7)	12,589 (90.3)	1,360 (9.7)	1.3 (1.2, 1.4), p<0.001
Age				
<18	3,868 (16)	3,159 (81.7)	709 (18.3)	1
18-49	14,716 (60.9)	1,3278 (90.2)	1,438 (9.8)	0.98 (0.88, 1.1) p=0.981
≥50	5,596 (23.1)	5,158 (92.2)	438 (7.8)	
Settlement type^*^				
Planned colony	6,494 (26.9)	5,848 (90.1)	646 (9.9)	1
Resettlement	2,627 (10.9)	2,359 (89.8)	268 (10.2)	(0.92, 1.1) p=0.76
Urban Slum	10,666 (44.1)	9,498 (89.0)	1,168 (11.0)	
Unauthorized	1,226 (5.1)	1,106 (90.2)	120 (9.8)	
Village	3,155 (13.1)	2,774 (87.9)	381 (12.1)	
History of COVID-19				
Present	6,662 (27.6)	6,048 (90.8)	614 (9.2)	0.98 (0.89, 1.1) p=0.754
Absent	17,506 (72.4)	15,537 (88.8)	1,969 (11.2)	1
Vaccine status				
Not taken (minor)	3,868 (15.9)	3,159 (81.7)	709 (18.3)	1
Not taken (adult)	6,605 (27.3)	5,418 (82.0)	1,187 (18.0)	
At-least one dose	13,760	13,065 (94.9)	695 (5.1)	4.2 (3.8, 4.6) p<0.001
One dose	6,033 (24.9)	5,737 (95.1)	296 (4.9)	
Two doses	7,727 (31.9)	7,328 (94.8)	399 (5.2)	

The seroprevalence was also comparable among the complete and partially vaccinated subgroups for both vaccines (Table 3). 

**Table 3:** Vaccination status and seroprevalence of antibodies to SARS-CoV-2, Delhi, September-October 2021 *A total of 13 participants had taken the Sputnik vaccine. Excludes <18 participants not eligible for vaccination. Missing vaccine status for 3,288 participants.

Vaccine type^*^	Total (n=13,760)	IgG Seropositive (95% CI)
Covishield 1 dose	5,140 (37.3)	4,914 (95.6) (95.0, 96.1)
Covishield 2 doses	6,086 (44.2)	5,802 (95.3) (94.8, 95.8)
Covaxin 1 dose	889 (6.5)	819 (92.1) (90.2, 93.7)
Covaxin 2 doses	1,628 (11.8)	1,514 (93.0) (91.6, 94.1)
History of COVID-19 present (n=6,662)		
Covishield 1 dose	1,603 (24.1)	1,541 (96.1) (95.1, 97.0)
Covishield 2 doses	2,095 (31.4)	2,006 (95.8) (94.8, 96.5)
Covaxin 1 dose	259 (3.9)	238 (91.9) (87.9, 94.6)
Covaxin 2 doses	554 (8.3)	516 (93.1) (90.7, 95.0)
No vaccination	2,143 (32.2)	1,739 (81.1) (79.4, 82.7)
No history of COVID-19 (n=17,506)		
Covishield 1 dose	3,537 (20.2)	3,373 (95.4) (94.6, 96.0)
Covishield 2 doses	3,991 (22.8)	3,796 (95.1) (94.4, 95.7)
Covaxin 1 dose	630 (3.6)	581 (92.2) (89.9, 94.1)
Covaxin 2 doses	1,074 (6.1)	998 (92.9) (91.2, 94.3)
No vaccination	8,265 (47.2)	6,781 (82.0) (81.2, 82.9)
Diabetes Mellitus (n=1,273)		
Covishield 1 dose	219 (17.2)	215 (98.2) (95.4, 99.3)
Covishield 2 doses	476 (37.4)	450 (94.5) (92.1, 96.2)
Covaxin 1 dose	70 (5.5)	68 (97.1) (90.2, 99.2)
Covishield 2 doses	174 (13.7)	158 (90.8) (85.6, 94.3)
No vaccination	313 (24.6)	262 (83.7) (79.2, 87.4)

The signal/cut-off (S/CO) of SARS-CoV-2 IgG in the seropositive samples (n=24,895) ranged from 1.00 to 22.8 (median 11.40, IQR 6.97, 14.5). A total of 21,620 (86.8%) seropositive individuals had a S/CO ≥4.0, signifying the likelihood of high antibody titres with the presence of neutralization antibodies. The proportion of SARS-CoV-2 IgG seropositive participants having a S/CO <4 was greater in females (14.2%) compared to male (12.4%) seropositive participants, and this difference was statistically significant (p<0.001). Furthermore, a S/CO <4 was observed in 29.1% of participants aged below 18 compared to only 11.1% and 9.2% in the 18-49 and ≥50 age-group seropositive participants, respectively (p<0.001).

From August 1, 2021 to October 26, 2021 (post second wave period), Delhi on average recorded daily 39 new cases (range 0 to 151), and 0.44 deaths (range 0 to 5). During the Omicron wave, from January 1, 2022 to March 31, 2022, Delhi on average recorded daily 4,267 cases (range 61 to 28,867), and 11.6 deaths (range 0 to 45) for a total of 413,793 positive cases and 1,045 deaths.

## Discussion

The findings of the sixth round of the serosurvey in Delhi indicate that nearly nine out of ten individuals aged five years and above in Delhi had detectable IgG SARS-CoV-2 antibodies which on assay adjustment reflect near-universal seropositivity. The seroprevalence in Delhi was higher compared to other Indian cities (58%-82%) but comparable with Mumbai (86.64%) probably because of the high severity of the second wave of the pandemic in these metropolitan cities [14-20].

The weighted and adjusted seroprevalence of IgG SARS-CoV-2 antibodies in Delhi increased from 50.1% and 56.1% in January 2021 to 88.1% and 97%, respectively, during September-October 2021 [7]. During the second wave of the countrywide COVID-19 pandemic, Delhi recorded nearly 737,000 cases including 11075 deaths [2]. Consequently, like the nationwide country pattern, the increase in seroprevalence was due to both natural infection and vaccination [21]. However, during this nine-month period, the seroprevalence increased in unvaccinated adults from 50.3% to 82%, and in children aged five years and above from 52.4% to 81.7%, which is indicative of natural infection being the primary driver of immune response in a large proportion of the population.

Similar to the trends in previous serosurvey rounds, the seroprevalence was found higher in adults compared to children and adolescents, in those having past- history of COVID-19, and in residents of planned colonies in comparison to other settlements. The seropositivity was also significantly higher in females compared to males despite lower rates of vaccination in the former suggesting a biological rather than behavioural phenomenon [6,7].

Previous studies suggest antibodies to SARS-CoV-2 persist at least one year after natural infection conferring durable protection against reinfection or symptomatic disease [22]. Unlike a previous study, the present study observed only a small difference in the rates of seroconversion between the Covishield and Covaxin vaccines [23]. Furthermore, the proportion of seronegative-vaccinated individuals in our study was very low even in those who had received only one dose of either of the vaccines. High seroprevalence was observed even in DM patients despite the possible association with an immunocompromised condition. However, in comparison, the nationwide ICMR study reported that 10%-14% of the vaccinated individuals remained seronegative even after receiving two vaccine doses [21]. This is possible because of the comparatively higher burden of natural infection in Delhi throughout the pandemic and later during the second wave. Moreover, there is growing recognition that even a single dose of COVID-19 vaccination post-infection induces a robust antibody response [24,25]. 

High S/CO suggestive of high antibody titres were observed in more than five of every six seropositive participants, while comparatively lower titres in the under-18 age group were likely because of their absence of vaccination. These findings indicate that a majority of the adults in Delhi had developed hybrid immunity due to COVID-19 vaccination after natural infection which is known to produce a robust immune response [26]. The small number of average daily COVID-19 cases and very low-test positivity rate after the second wave subsiding in Delhi is suggestive of the attainment of the herd immunity threshold in the population with the caveat for the expected waning of antibody titres over time [27,28].

The robust antibody response from natural immunity and vaccination translated into effective population protection evident from the low number of cases and deaths after the cessation of the second wave. Subsequently, the Omicron variant triggered the third wave of the pandemic in Delhi by frequently bypassing existing immunity although the low number of deaths in the wave correlates with the presence of hybrid immunity in a majority of the adult population in Delhi that conferred them protection against severe disease, hospitalization, and death [29]. Precautionary or booster doses were permitted for adults and comorbid only from January 2022, which suggests existing hybrid immunity was the major cause of immune protection until then.

The study has certain limitations. First, due to duplicate unique id generated by field data enumerators and failure of the android application's validated check, the questionnaire data of ~10% participants could not be matched with the unique identification labels of their laboratory samples resulting in a loss of their sociodemographic attributes (except age and sex attributes recovered from laboratory data) and vaccination status. During analysis, this missing data were assumed to be missing at random type. Second, information about the non-responding households was not collected in the data collection application by the field volunteers precluding the possibility of verifying the extent of non-response, although based on consultation with field workers and nodal officers it was expected to be not dissimilar from the previous round (<20%). Third, in this investigation, we could not differentiate between vaccination or natural infection-induced antibody response since both anti-S and anti-N antibodies are generated by inactivated vaccines such as Covaxin [11]. Fourth, the S/CO only provided an indirect marker of IgG SARS-CoV-2 antibody titres and was not an absolute correlate of immunological protection. Moreover, immune protection against SARS-CoV-2 can be triggered through both humoral and cell media immunity, and consequently, seropositivity may not necessarily be indicative of protection against COVID-19 disease or vice versa [11,30]. Finally, the participant’s vaccination status and COVID-19 disease history were often ascertained from individual recall in absence of validation with the vaccination certificate and laboratory record, respectively.

## Conclusions

The findings of this serosurvey indicate the presence of IgG antibodies against SARS-CoV-2 in most of the general population of Delhi, although the seroprevalence in the vaccinated group was higher compared to the unvaccinated group, especially in those without a past history of COVID-19 disease. Consequently, vaccination coverage needs acceleration in the unvaccinated and those partially vaccinated with only one dose of COVID-19 vaccine, especially for protection against severe disease and death. Continuous genomic surveillance, pandemic preparedness, and adherence to non-pharmaceutical interventions without any laxity or complacence require high and sustained prioritization to diminish morbidity and mortality from future waves of the pandemic.
